# Socioeconomic inequalities in the prevalence, non-awareness, non-treatment, and non-control of diabetes among South Korean adults in 2021

**DOI:** 10.1371/journal.pone.0313988

**Published:** 2024-11-21

**Authors:** Seongju Kim, Dong Jun Kim, Hooyeon Lee

**Affiliations:** 1 Department of Public Health and Healthcare Management, Graduate School, The Catholic University of Korea, Seoul, Korea; 2 Department of Preventive Medicine, College of Medicine, The Catholic University of Korea, Seoul, Korea; 3 Department of Public Health, Graduate School, The Catholic University of Korea, Seoul, Korea; Kyung Hee University School of Medicine, REPUBLIC OF KOREA

## Abstract

The purpose of this study was to investigate socioeconomic inequalities in diabetes prevalence, non-awareness, non-treatment, and non-control among South Korean adults in 2021. This cross-sectional study used data from the 2021 Korean National Health and Nutrition Examination Survey. Relative concentration indices (RCIs) and relative concentration curves stratified by sex and age were used to investigate socioeconomic inequalities in the prevalence, non-awareness, non-treatment, and non-control of diabetes. The prevalence, non-awareness, lack of treatment, and non-control rates in adults aged 30 years and older in 2021 were 15.9%, 29.5%, 33.3%, and 76.1%, respectively. Diabetes was more prevalent in participants under the age of 65 years than those aged 65 years and older for both men (RCI: -0.081, RCI: -0.158, respectively) and women (RCI: -0.203, RCI: -0.292, respectively). The larger the absolute value of the RCI in non-awareness and non-treatment of diabetes in women, the greater the level of socioeconomic inequalities (RCI: 0.182, RCI: 0.154). Socioeconomic inequalities existed in the prevalence of diabetes among both men and women aged under 65 years. In women, socioeconomic inequalities of non-awareness and non-treatment of diabetes were greater than those in men. Thus, preventive care and monitoring are required, particularly among women and individuals under the age of 65 years.

## Introduction

Diabetes mellitus (DM) is a common and major global health problem that leads to various complications and organ dysfunction, such as cardiovascular disease, nephropathy, and blindness [1]. DM is associated with long-term threats that significantly increase individual and national socioeconomic burdens [2]. The global prevalence of DM in adults aged 20–79 years has increased from 9.3% in 2019 to 10.5% in 2021 [3]. In South Korea, the prevalence of DM among adults aged over 30 years increased from 14.1% in 2010–2012 [4] to 16.7% in 2020 [5].

A substantial proportion of patients with DM is unaware of their diabetes status and blood glucose levels owing to the silent nature of DM [6, 7]. In South Korea, 35% of adults with DM are undiagnosed and only 25% of patients with DM achieve the target HbAlc level of less than 6.5% [3]. Non-control of DM is associated with hemorrhagic stroke and cardiovascular and kidney diseases. Thus, adequate awareness and prerequisites for optimal treatment compliance are important for the successful control of DM [4].

Socioeconomic status has been operationalized in various ways, most commonly as education or income. The relationship between education and health is multifaceted, influenced by various socioeconomic factors [8]. Education serves as a crucial bridge across generations, impacting health through several mechanisms, including human capital development, psychosocial resources, and lifestyle choices. Income is often considered a straightforward indicator of material resources, and is, robustly and positively, associated with longevity [9].

Socioeconomic disparities influence follow-up at multiple levels, including access to healthcare, process of care, and individual patient capabilities [10]. These can lead to a lack of awareness of DM or willingness to receive treatment for DM [11]. Therefore, monitoring the relationship between SES and diabetes is imperative for the development of targeted interventions aimed at management of DM and improving quality of life [4, 10–13].

Clinical guidelines for DM prevention and management emphasize the importance of individual awareness and its risk factors [3, 11]. However, previous studies in South Korea have mainly focused on the prevalence of DM, whereas studies on non-awareness, non-treatment, and non-control of DM are lacking. The increasing prevalence of DM will considerably affect the socioeconomic burden over time [12]. Therefore, we aimed to identify the socioeconomic inequalities of patients with type 2 DM among South Korean adults aged over 30 years using data from a recent nationally representative survey.

## Methods

### Research data and participants

We used data from the 2021 Korean National Health and Nutrition Examination Survey (KNHANES). The KNHANES is a national cross-sectional survey conducted by the Korea Centers for Disease Control and Prevention, designed to assess the health-related behaviors, health, and nutritional status of the South Korean population. Based on the Population and Housing Census, 192 primary sampling units with 4,800 households were selected using stratified multistage probability sampling to represent the entire South Korean population. Family members over the age of 1 year selected primary sampling units and households as subjects for sampling, corresponding to approximately 10,000 individuals [14, 15].

We analyzed data from adults aged over 30 years following prior DM studies [16–18]. We excluded individuals under 30 years old because this group is likely to be socioeconomically unstable, especially in terms of education and income status. Therefore, we focused on adults aged 30 years and older. In total, 4,324 participants, including 1,886 (49.6%) men and 2,438 (50.4%) women, were included after excluding those with missing data. Among the total participants, 801 (15.9%) had type 2 DM. All participants voluntarily participated in the health examination and health interview and provided written informed consent. This study was approved by the Institutional Review Board (IRB) of the College of Medicine, the Catholic university of Korea (approval number: MC24ZASI0111).

### Dependent variables

The prevalence of type 2 DM was determined based on the following criteria: (1) a diagnosis of DM by a doctor, (2) fasting plasma glucose level ≥ 126 mg/dL, (3) taking oral hypoglycemic agents or insulin injections, or (4) HbA1C level of ≥ 6.5% according to the criteria set by the Korean Diabetes Association [13]. Awareness of type 2 DM was assessed by asking “Have you been diagnosed with diabetes by a doctor?” Participants who answered “no” with DM were categorized as being unaware of DM [19]. Non-treatment of type 2 DM was defined as participants with DM who were not receiving treatment for DM with oral anti-diabetic drugs or insulin injections. Non-control of type 2 DM was defined as having an HbA1C level ≥ 6.5% in patients with DM [13].

### Household income and education level

We used household income and education level as socioeconomic indicators. For equivalized households, monthly income was calculated by dividing the square root of household members and categorizing it into five groups from the lowest (Q1) to the highest (Q5) quintile. Education level was categorized as below elementary school or graduate, junior high, high school graduate, and college graduate or higher.

### Covariates

Demographic factors included age, region, and health indicators, including waist circumference, body mass index (BMI), smoking status, drinking status, and family history of DM. We categorized the region as urban (administrative divisions of a city) or rural (living in a town or in the countryside) [20, 21]. Waist circumference was classified as < 90 and ≥ 90 cm in men and < 80 and ≥ 80 cm in women. BMI was calculated as weight (kg) divided by height in meters squared and classified into the three groups of < 23.0, 23.0–24.9, and ≥ 25.0 using the Asia-Pacific criteria [22]. Current smoking was categorized as “no” or “yes” if the participants had smoked 100 cigarettes in their lifetime. Current drinking was categorized as “no” or “yes,” with alcohol intake of more than once per month during the past 12 months. We defined family history of DM as having a first-degree relative (parents or siblings) with DM [23], which was categorized as “no” or “yes”.

### Socioeconomic inequality

We used the relative concentration index (RCI) and relative concentration curve (RCC) to investigate socioeconomic inequalities in diabetes. The RCI is a bivariate measure that examines the distribution of a health variable and the socioeconomic status ranks [24]. The RCC plotted the cumulative percentage of ill-health on the y-axis against the cumulative percentage of the population ranked by socioeconomic group status on the x-axis. The RCI is derived from the RCC [25]. If the curves were above the equality line (RCI < 0), the prevalence, non-awareness, non-treatment, and non-control of DM were more common in the lowest than highest groups. If the curves were below the equality line (RCI > 0), the prevalence, non-awareness, non-treatment, and non-control of DM were more common in the highest than lowest income groups. Additionally, the larger the absolute value of the RCI, the greater the socioeconomic disparity [25, 26].

### Statistical analysis

Descriptive statistics were calculated to determine the sociodemographic characteristics regarding prevalence, non-awareness, non-treatment, and non-control rates of type 2 DM by sex and age using analysis methods for complex sampling data. The characteristics were calculated as weighted percentages for the categorical variables. To compare the proportions of the variables, we conducted a chi-squared test for the categorical variables.

Complex sample analyses were performed using population weights, and the details of weights were provided by the KNHANES (https://knhanes.kdca.go.kr/knhanes/main.do/). All statistical analyses were conducted using STATA MP 18 (STATA Corp., College Station, TX, USA) [27, 28]. For all test, two-sided p < 0.05 indicated statistical significance.

## Results

Tables 1 and 2 provide the weighted prevalence, non-awareness, non-treatment, and non-control rates of DM by sex. The prevalence rate of DM for adults aged > 30 years was 15.9%. Prevalence rates by sex were 18.9% for men and 13.0% for women. Non-awareness rates were 29.5% for the total subjects, 29.7% for men, and 29.3% for women. Non-treatment rates were 33.3% for the total subjects, 33.9% for men, and 32.4% for women. Non-control rates were 76.1% for the total subjects, 76.2% for men, and 75.9% for women. The prevalence rates were higher for the lowest income group (men: 31.3%, women: 32.1%) and lowest education group (men: 26.5%, women: 30.3%). However, the non-awareness and non-treatment rates were higher for the highest income group and highest education group in both men and women.

**Table 1:** Weighted prevalence, non-awareness, non-treatment, and non-control rates of type 2 diabetes among men in 2021 in South Korea (n = 1,886).

Variables	Prevalence rate		Non-awareness rate		Non-treatment rate		Non-control rate	
	n	%	n	%	n	%	n	%
Total	413	18.9	125	29.7	145	33.9	306	76.2
Age								
30≤ <65	209	15.5	73	31.7	83	36.0	157	77.5
≥ 65	204	32.8	52	25.7	62	29.8	149	73.8
Equivalized household income								
Q5	72	15.0	28	37.1	31	40.2	53	75.2
Q4	48	15.2	14	25.0	15	26.9	38	84.9
Q3	86	17.5	25	32.2	32	39.2	62	71.2
Q2	99	22.2	30	27.8	35	33.0	75	79.5
Q1	108	31.3	28	24.1	32	26.4	78	73.5
Education level								
≥ College	133	13.4	44	30.7	49	34.3	102	77.1
High School	140	23.0	42	30.0	49	34.6	103	77.0
Junior High	59	28.5	17	29.1	20	33.2	40	69.7
≤ Elementary	81	32.1	22	26.0	27	31.3	61	77.4
Region								
Urban	300	18.4	94	30.6	105	34.1	228	78.0
Rural	113	20.8	31	25.9	40	33.2	78	69.3
Waist circumference								
< 90 cm	173	13.7	50	25.0	59	29.3	124	74.1
≥ 90 cm	240	25.7	75	33.0	86	37.2	182	77.8
BMI								
< 23.0	97	15.3	23	23.7	27	26.7	67	72.1
23.0–24.9	100	15.3	24	17.6	28	22.1	73	71.9
≥ 25.0	216	23.0	78	36.6	90	41.3	166	79.6
Current smoking								
No	295	18.4	93	30.8	103	33.7	216	75.0
Yes	118	20.1	32	27.2	42	34.3	90	79.1
Current drinking								
No	169	22.6	36	23.7	44	26.8	124	75.7
Yes	244	17.1	89	33.4	101	38.4	182	76.6
Family history of DM								
No	255	15.0	87	32.9	102	37.3	182	74.3
Yes	158	30.1	38	25.0	43	29.1	124	79.0

Notes: Quintile 1 (Q1) is the lowest and quintile 5 (Q5) is the highest; BMI, body mass index; DM, diabetes mellitus.

**Table 2:** Weighted prevalence, non-awareness, non-treatment, and non-control rates of type 2 diabetes among women in 2021 in South Korea (n = 2,438).

Variables	Prevalence rate		Non-awareness rate		Non-treatment rate		Non-control rate	
	n	%	n	%	n	%	n	%
Total	388	13.0	103	29.3	117	32.4	294	75.9
Age								
30≤ <65	153	8.2	51	38.3	58	42.0	122	78.6
≥ 65	235	29.0	52	21.0	59	23.5	172	73.4
Equivalized household income								
Q5	32	5.9	12	35.4	15	43.1	21	64.7
Q4	29	9.1	12	43.1	14	47.9	23	76.6
Q3	76	10.5	20	27.1	21	29.0	57	77.3
Q2	100	15.6	26	35.3	28	36.9	79	80.8
Q1	151	26.5	33	19.4	39	22.5	114	74.0
Education level								
≥ College	55	5.2	17	34.2	23	44.1	39	70.0
High School	97	12.3	36	42.7	38	44.1	75	78.2
Junior High	67	21.2	17	27.5	18	28.6	56	84.0
≤ Elementary	169	30.3	33	17.2	38	19.3	124	73.5
Region								
Urban	268	11.7	73	30.2	81	32.8	207	75.8
Rural	120	19.9	30	26.7	36	31.0	87	76.1
Waist circumference								
< 80 cm	81	5.6	18	26.8	23	31.3	52	66.3
≥ 80 cm	307	20.5	85	30.0	94	32.7	242	78.6
BMI								
< 23.0	108	7.9	19	20.1	27	25.6	71	67.2
23.0–24.9	76	10.8	11	13.0	13	14.6	57	73.7
≥ 25.0	204	22.0	73	39.0	77	41.2	166	81.2
Current smoking								
No	369	13.1	95	28.1	109	31.3	281	76.5
Yes	19	11.7	8	56.7	8	56.7	13	63.6
Current drinking								
No	302	15.5	72	26.5	82	29.2	230	77.8
Yes	86	8.9	31	37.5	35	41.7	64	70.3
Family history of DM								
No	226	10.7	67	34.1	77	38.3	166	75.2
Yes	162	18.6	36	22.5	40	24.1	128	76.9

Notes: Quintile 1 (Q1) is the lowest and quintile 5 (Q5) is the highest; BMI, body mass index; DM, diabetes mellitus.

Tables 3 and 4 present the RCIs by sex stratified by age for the prevalence, non-awareness, non-treatment, and non-control of type 2 DM according to income and education. Type 2 DM was more prevalent in low SES groups than high SES groups for both men and women (RCI in men: 0.131, RCI in women: 0.177). As shown in Table 3, the absolute values of the income and education RCIs were higher for men under the age of 65 years (RCI: -0.081, -0.158) than for those aged 65 years and older (RCI: -0.040, 0.008). However, socioeconomic inequalities regarding non-awareness, non-treatment, and non-control among men with DM were not significant for age. With regard to women (Table 4), the absolute values of the income RCIs for non-awareness and non-treatment of DM were higher for those over the age of 65 years (RCI: 0.182, 0.154). However, socioeconomic inequalities regarding non-control of DM were not significant.

**Table 3:** Relative concentration indices for the prevalence, non-awareness, non-treatment, and non-control of type 2 diabetes in men.

Variables	Total			Age 30≤ <65			Age ≥ 65		
	RCI	SE	p-value	RCI	SE	p-value	RCI	SE	p-value
**Equivalized household income**									
Prevalence	0.131	0.027	0.000	0.081	0.038	0.036	0.040	0.035	0.261
Non-awareness	0.064	0.047	0.174	0.026	0.056	0.644	0.091	0.066	0.170
Non-treatment	0.053	0.040	0.186	0.005	0.048	0.912	0.106	0.059	0.077
Non-control	0.006	0.017	0.723	0.001	0.020	0.978	0.003	0.026	0.910
**Education level**									
Prevalence	0.177	0.028	0.000	0.158	0.036	0.000	0.008	0.041	0.847
Non-awareness	0.022	0.044	0.616	0.012	0.052	0.823	0.002	0.065	0.976
Non-treatment	0.012	0.040	0.774	0.003	0.048	0.949	0.021	0.065	0.745
Non-control	0.007	0.017	0.677	0.009	0.022	0.688	0.011	0.027	0.677

Notes: RCI, relative concentration index; SE, standard error.

**Table 4:** Relative concentration indices for the prevalence, non-awareness, non-treatment, and non-control of type 2 diabetes in women.

Variables	Total			Age 30≤ <65			Age ≥ 65		
	RCI	SE	p-value	RCI	SE	p-value	RCI	SE	p-value
**Equivalized household income**									
Prevalence	0.272	0.033	0.000	0.203	0.050	0.000	0.057	0.037	0.129
Non-awareness	0.116	0.045	0.011	0.056	0.056	0.323	0.182	0.075	0.017
Non-treatment	0.119	0.044	0.007	0.028	0.054	0.604	0.154	0.067	0.025
Non-control	0.007	0.018	0.719	0.044	0.024	0.076	0.009	0.025	0.725
**Education level**									
Prevalence	0.358	0.029	0.000	0.292	0.055	0.000	0.048	0.030	0.115
Non-awareness	0.171	0.046	0.000	0.090	0.059	0.134	0.093	0.083	0.263
Non-treatment	0.187	0.044	0.000	0.127	0.057	0.030	0.091	0.077	0.239
Non-control	0.002	0.018	0.923	0.024	0.025	0.332	0.002	0.023	0.928

Notes: RCI, relative concentration index; SE, standard error.

**Fig 1:**
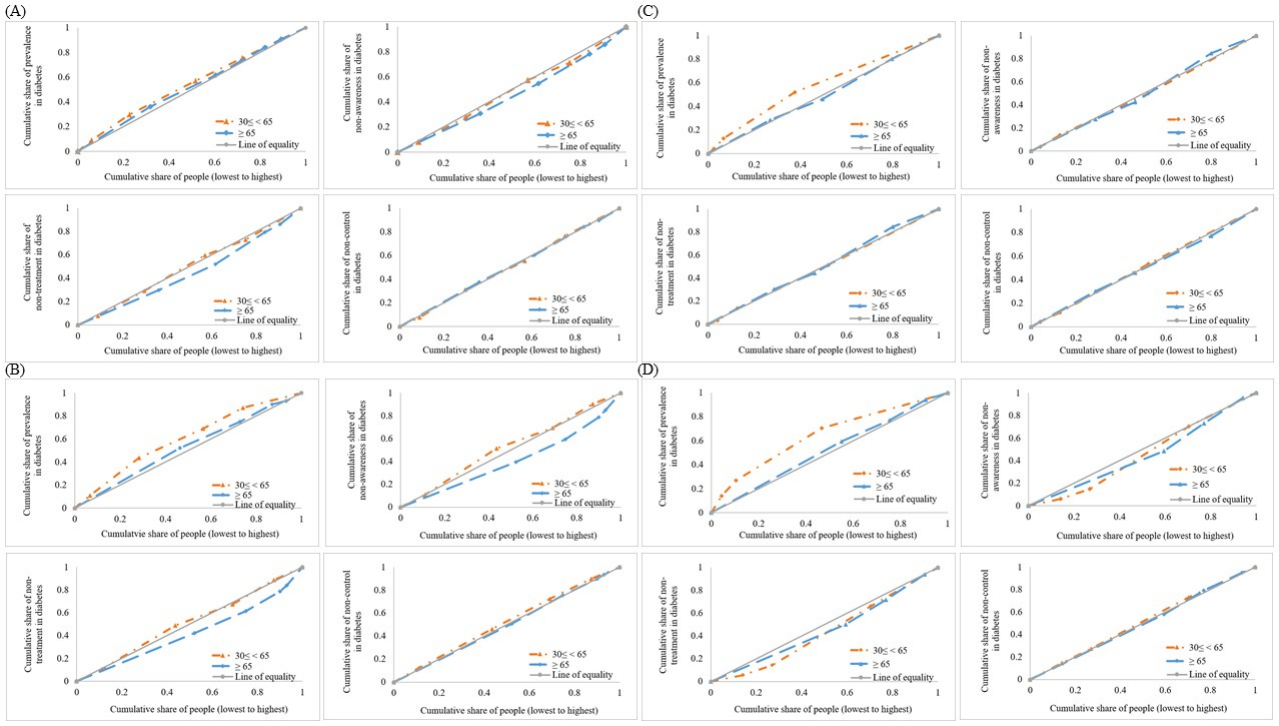
Relative concentration curves for the prevalence, non-awareness, non-treatment, and non-control of diabetes by sex and age. (A) Income-related inequalities in men, (B) Income-related inequalities in women, (C) Education-related inequalities in men, (D) Education-related inequalities in women.

The RCCs for socioeconomic inequalities by sex stratified by age are shown in Fig 1. For both men and women, the RCCs for prevalence of DM were above the line of equality, which implies socioeconomic inequality in the lowest group for DM in South Korea. However, the RCCs for the non-control of DM were at the equality line, indicating that there was no socioeconomic inequality for both men and women.

## Discussion

### Main findings

This study investigated the prevalence, non-awareness, non-treatment, and non-control rates of DM and socioeconomic inequality using the 2021 KNHANES as representative national data. The prevalence of DM among the 4,324 participants was 15.9%, and the rate for men (18.0%) was higher than that for women (13.0%). This proportion is higher than the 13.9% reported in a previous study of South Koreans in 2020 [3] and 8.7% in Japan [29].

Socioeconomic inequalities of DM were prevalent in the lowest income and education groups for both men and women. Several studies have reported an association between low SES and prevalence of DM [4, 7, 29]. People of low SES are exposed to high-risk factors of DM and to more unfavorable living conditions [29, 30]. We found the higher the value of the RCI for prevalence of DM, the greater the socioeconomic inequalities for those under the age of 65 years in both men and women. This may be explained by the association of DM with aging; DM in people aged ≥ 65 years may be more influenced by physical status rather than external factors such as income and level of education [31, 32].

The non-awareness, non-treatment, and non-control rates of DM were 29.7%, 33.3%, and 76.0%, respectively. Compared with other studies conducted in South Korea, the non-awareness rate was 26.4% in 2015 [4] and non-treatment rate was 38.4% in 2019 [33]. Studies conducted in other countries have reported rates for poor glycemic control of DM ranging from 50.2 to 76.0% [7, 34–37]. Non-awareness and inadequate control reflect low opportunities for reducing the growing global burden of DM [4, 25]. Furthermore, non-control of DM causes disability and shortens lives. As such, continuity of care is of crucial importance.

### Socioeconomic inequalities

In our study, socioeconomic inequalities in non-awareness and non-treatment of DM were found for women, but not for men. In South Korea, people can check their DM status every two years through a universal health screening program so that they can be aware of their DM status [4, 14]. However, the participation rates have been slightly lower in women (72.6%) than men (75.6%) [38]. Moreover, higher involvement from women in child rearing and homemaking may act as a hindrance to seeking medical services or treatment for themselves, especially in low-income households [39, 40].

Income-related inequalities in non-awareness and non-treatment of DM were higher in the highest groups than lowest groups. In 2016 in South Korea, a community-based primary care project introduced a pilot program for DM care to improve the efficiency of DM management [41]. A study reported that the greater inequality of DM in personal medical expenses in the high income group and medical service utilization increased among low income groups [42]. It may be seen that the obstacles of utilization services being reduced for patients of low SES. However, in the case of the medical expenditure when using medical services for the same medical needs, the burden on the lower income group would be greater than the high income group. Education-related inequalities showed similar patterns with income in our study. This may be explained by the close association between income and level of education [43]. Thus, policies need to be implemented to decrease the burden of medical expenditures for those with low SES.

Unexpectedly, we did not observe socioeconomic inequalities in non-control of DM for men or women regardless of age. However, this result coincides with other studies that found no significant difference in non-control of DM according to SES [4, 44]. Other findings have indicated that control of DM was more related to social factors, such as guidelines or protocols of management, access to healthcare utilization, and support for the treatment of DM, than income and educational factors [45]. Additionally, different ranges in HbA1C level of controlled DM can have a long-term effect on glycemic control [46–48]. Therefore, future research is needed considering glycemic control of DM with various social and socioeconomic factors.

### Implications for research

Our study suggests that DM prevention and monitoring strategies should focus on prevalence and management as functions of socioeconomic position. DM has been proposed as a good tracer condition that can provide insights into the national healthcare system [49]. This study also suggests that early interventions should include primary care efforts targeted at women and adults aged under 65 years to reduce disparities. Monitoring may emphasize the gaps in preventive and care services supplied to vulnerable individuals and may persuade governments and physicians to address these issues [50].

This study has several limitations. First, this was a cross-sectional study, making it difficult to determine the causal relationship between SES and DM because of the possibility of reverse causality. Further research is needed to examine trends in socioeconomic inequalities associated with diabetes in South Korea. Second, we were unable to determine the type of DM using data from the KNHANES. Therefore, we confined the participants to those aged over 30 years to reduce the possibility of including patients with type 1 DM. Third, a fasting glucose test performed on a single day may not be indicative of having DM.

## Conclusion

This study observed socioeconomic inequalities in the prevalence, non-awareness, lack of treatment, and non-control rate of DM using nationally representative data in South Korea. It highlights the insufficient management of DM and income and education-related inequalities among women and people under the age of 65 years with DM. With the increasing prevalence of DM in South Korea, SES is significantly affecting lifestyles, leading to deficiencies in the organization and quality of medical care. Therefore, monitoring socioeconomic inequalities, particularly among women and people under the age of 65 years, is necessary to establish DM prevention and management policies.
